# Cocaine use associated gut permeability and microbial translocation in people living with HIV in the Miami Adult Study on HIV (MASH) cohort

**DOI:** 10.1371/journal.pone.0275675

**Published:** 2022-10-10

**Authors:** Jacqueline Hernandez, Javier A. Tamargo, Sabrina Sales Martinez, Haley R. Martin, Adriana Campa, Rafick-Pierre Sékaly, Rebeka Bordi, Kenneth E. Sherman, Susan D. Rouster, Heidi L. Meeds, Jag H. Khalsa, Raul N. Mandler, Shenghan Lai, Marianna K. Baum

**Affiliations:** 1 Robert Stempel College of Public Health and Social Work, Florida International University, Miami, Florida, United States of America; 2 Department of Pathology and Laboratory Medicine, Emory University School of Medicine, Atlanta, Georgia, United States of America; 3 Division of Digestive Diseases, Department of Internal Medicine, University of Cincinnati College of Medicine, Cincinnati, Ohio, United States of America; 4 Department of Microbiology, Immunology and Tropical Diseases, George Washington University School of Medicine and Health Sciences, Washington, DC, United States of America; 5 National Institute on Drug Abuse, Rockville, Maryland, United States of America; 6 Department of Epidemiology, Institute of Human Virology, University of Maryland School of Medicine, Baltimore, Maryland, United States of America; Tulane National Primate Research Center, UNITED STATES

## Abstract

**Objective:**

Determine if cocaine use impacts gut permeability, promotes microbial translocation and immune activation in people living with HIV (PLWH) using effective antiretroviral therapy (ART).

**Methods:**

Cross-sectional analysis of 100 PLWH (ART ≥6 months, HIV-RNA <200 copies/mL) from the Miami Adult Studies on HIV (MASH) cohort. Cocaine use was assessed by self-report, urine screen, and blood benzoylecgonine (BE). Blood samples were collected to assess gut permeability (intestinal fatty acid-binding protein, I-FABP), microbial translocation (lipopolysaccharide, LPS), immune activation (sCD14, sCD27, and sCD163) and markers of inflammation (hs-CRP, TNF-α and IL-6). Multiple linear regression models were used to analyze the relationships of cocaine use.

**Results:**

A total of 37 cocaine users and 63 cocaine non-users were evaluated. Cocaine users had higher levels of I-FABP (7.92±0.35 vs. 7.69±0.56 pg/mL, P = 0.029) and LPS (0.76±0.24 vs. 0.54±0.27 EU/mL, P<0.001) than cocaine non-users. Cocaine use was also associated with the levels of LPS (P<0.001), I-FABP (P = 0.033), and sCD163 (P = 0.010) after adjusting for covariates. Cocaine users had 5.15 times higher odds to exhibit higher LPS levels than non-users (OR: 5.15 95% CI: 1.89–13.9; P<0.001). Blood levels of BE were directly correlated with LPS (rho = 0.276, P = 0.028), sCD14 (rho = 0.274, P = 0.031), and sCD163 (rho = 0.250, P = 0.049).

**Conclusions:**

Cocaine use was associated with markers of gut permeability, microbial translocation, and immune activation in virally suppressed PLWH. Mitigation of cocaine use may prevent further gastrointestinal damage and immune activation in PLWH.

## Introduction

People living with HIV (PLWH) exhibit pathological processes that affect the gastrointestinal tract. One of the main features of infection with the human immunodeficiency virus (HIV) is the massive destruction of CD4+ T cells in the gastrointestinal tract, which are more frequently infected by HIV than those in blood, indicating the gastrointestinal tract as a major target and viral reservoir for HIV in the human body [1]. The loss and poor reconstitution of CD4+ T cells in the gastrointestinal tract promote disruption of intestinal homeostasis, mucosal regeneration, and gut integrity despite antiretroviral therapy (ART) [2–6].

Preserving the gut barrier is vital for the intestinal epithelium. When the intestinal barrier is damaged, intestinal permeability increases, allowing translocation of products including lipopolysaccharide (LPS), into the bloodstream, causing endotoxemia and inflammation [7]. I-FABP are small cytosolic proteins that transport fatty acids to the enterocytes. In the event of intestinal ischemia or gut integrity damage, I-FABP is leaked from the gut to the systemic circulation indicating intestinal barrier dysfunction and permeability [8].

The diversity of the intestinal microbiome appears to be reduced in PLWH [9–15]. The combination of reduced intestinal CD4+ T cells and alterations in the microbiome contribute to disruptions of tight junctions in the intestinal epithelium, which in turn leads to the loss of gut integrity and microbial translocation in PLWH [16]. Microbial translocation (MT) refers to the passage or transport of the microflora or its products from the gastrointestinal tract into the systemic circulation without causing bacteremia [17]. PLWH present with products of MT; specifically LPS, also known as endotoxin [18, 19]. In turn, LPS in the circulation induces immune activation and inflammation, considered hallmarks of HIV infection and predictors of poor clinical outcomes in PLWH [20–24]. Chronic immune activation has been identified as one of the factors that increase morbidity and mortality among PLWH regardless of ART use and viral suppression and [25]. In addition, constant low levels of viral replication from viral reservoirs, particularly in the gastrointestinal tract, persist despite effective ART thereby promoting chronic immune activation [26]. This creates a vicious cycle where gut barrier immunity is compromised by HIV infection, promoting microbial translocation and immune activation, which in turn contribute to HIV disease progression.

Cocaine is one of the most frequently used illicit drugs in the United States [27] and it is disproportionately used by PLWH [28]. Epidemiological studies have demonstrated that active illicit drug use, particularly cocaine, is associated with poor adherence to ART, reduced virologic and immunologic control, HIV disease progression, and mortality [29, 30]. Cocaine use poses deleterious risks related to the gastrointestinal tract, including malnutrition, anorexia, gastrointestinal bleeding, reduced blood supply to enterocytes, and dysbiosis of the gut microbiome among PLWH [31–33]. Also, in vitro and in vivo studies suggest that cocaine can impair gut integrity, leading to microbial translocation and systemic inflammation [34, 35]. Nonetheless, the role of cocaine use on gut permeability, microbial translocation, and immune activation among PLWH has not been thoroughly investigated and characterized. Therefore, the purpose of this study was to examine the relationships between cocaine use, gut permeability, microbial translocation, and immune activation in the context of HIV infection.

## Methods

### Study participants

Participants for this cross-sectional study were selected from the ongoing Miami Adult Studies in HIV (MASH) cohort, a National Institute on Drug Abuse (NIDA)-funded study examining the impact of substance use on HIV infection and comorbidities. The MASH cohort includes a large number of non-Hispanic Blacks, Hispanics, and non-injection drug users who are followed every 6 months with comprehensive surveys administered on the same day as fasting blood and urine samples are collected. Participants for this study were selected from the MASH cohort if they met the following criteria: age 21 and older; HIV infected on ART with suppressed HIV viral load (<200 copies/ml) and CD4+ T cell count >200 cells/μL for at least 6 months; and no hazardous drinking as determined by an Alcohol Use Disorders Identification Test (AUDIT) score below 8. The exclusion criteria for this study were comprised of co-infection with hepatitis B or C virus and diagnosis of an inflammatory bowel disease, confirmed with review of medical records. This study was approved by the Florida International University Institutional Review Board. All participants provided written consent for participation in the study and release of medical records.

### Laboratory analyses

Blood samples were collected every 6 months as part of each MASH cohort study visit and aliquoted for storage at -80° for later use. Plasma samples collected at baseline were used to assess blood levels of LPS for microbial translocation, intestinal fatty-acid binding protein (I-FABP) a biomarker for gut integrity, soluble CD14 (sCD14), sCD163, and sCD27 for immune activation, and hs-CRP, TNF-α and IL-6 for inflammation. I-FABP was assessed using the Human FABP2/I-FABP Immunoassay by R&D Systems (Minneapolis, MN, USA) according to manufacturer’s instructions with a sample dilution of 1:5. LPS was measured using the Pierce Chromogenic Endotoxin Quant Kit by Thermo Fisher Scientific (Rockford, IL, USA) according to manufacturer’s instructions with the following modifications: samples were diluted 1:10 with endotoxin-free water to avoid interference with background color and preheated to 70°C for 15 minutes prior to analyses to inactivate plasma proteins. Triplicates were assessed for each sample. Immune activation markers were measured in singlicate using the magnetic analyte-specific bead-based Luminex multiplex immunoassays (Luminex Human Discovery Assays, R&D Systems, Minneapolis, MN, USA). Soluble CD14 was assayed at a 1:200 plasma dilution, while sCD27, and sCD163 were run at 1:2. Analyte concentrations were determined by the protocol-based data acquisition software using a standard curve derived from the known reference concentration as supplied by the manufacturer. TNF-α and IL-6 were measured by using the analyte-specific bead-based Luminex multiplex immunoassays (EMD, Millipore Corporation). High-sensitivity C-reactive protein (hs-CRP), a marker of systemic inflammation, was also measured in blood (Labcorp, Burlington, NC, USA).

### Substance use

Cocaine use was defined as self-reported use (in the past 30 days), a positive urine drug screen (American Bio Medica^®^, Kinderhook, NY, USA), or blood benzoylecgonine (BE) concentration ≥0.1 ng/mL. Cocaine is rapidly converted in the liver into its primary metabolite, BE [36]. A modified liquid chromatography with tandem mass spectrometry (LC-MS/MS) method was used to measure BE concentrations in plasma [37]. The target analyte and internal standard were identified and quantified using a triple quadrupole mass spectrometer (TSQ Quantum Ultra with a HESI-II probe, Thermo Fisher Scientific Inc., San Jose, CA, USA). Cigarette smoking and alcohol consumption (AUDIT score) was self-reported.

### HIV disease progression and medication history

Markers of HIV disease progression (CD4+ T cell count and HIV viral load) were abstracted from medical records with participants’ written authorization. Use and adherence with ART was obtained from the AIDS Clinical Trials Group (ACTG) validated adherence questionnaire administered to study participants.

### Statistical analyses

Descriptive statistics including means, standard deviations, and percentages were used to describe the data. Log transformations were performed for non-normally distributed continuous outcomes for I-FABP, LPS, sCD14, sCD27, sCD163, hs-CRP, TNF-α and IL-6. Independent sample T-tests (or non-parametric Wilcoxon Rank-Sum Test) were conducted to compare the distribution of continuous outcomes between cocaine users and non-users. Pearson and Spearman correlations were used to evaluate correlations between continuous variables. Multiple linear regression models were implemented to analyze the relationships of cocaine use with log-transformed I-FABP, LPS, sCD14, sCD27, sCD163, hs-CRP, TNF-α and IL-6 while controlling for covariates [age, sex, race, CD4 cell count, suppressed HIV viral load (20–200 copies/mL), and cigarette smoking]. The dependent variables in the logistic regression models were I-FABP and LPS at the following cut-offs: I-FABP and LPS ≤ or ≥ median. An alpha less than 0.05 was considered statistically significant. All statistical analyses were performed using SPSS software, version 27.

## Results

### Participant characteristics

A total of 100 PLWH were selected for this study, with mean age of 53.4±6.9 years (Table 1). Thirty-seven participants (37%) used cocaine. Most participants were African American (66%) and 29% were Hispanic. Participants had a mean duration of known HIV diagnosis of 17.2±7.3 years, 78% had undetectable HIV viral load (<20 copies/mL) and 22% were virally suppressed (HIV viral load 20–200 copies/mL). Participants had a mean BMI of 28.7±5.8 kg/m^2^ and 37% were obese. Approximately twice as many cocaine users smoked cigarettes than cocaine non-users (64.9% vs. 36.5%, P = 0.006).

**Table 1:** Participant’s characteristics^a^.

Characteristics	Total	Cocaine Non-users	Cocaine Users	P value^b^
	(N = 100)	(N = 63)	(N = 37)	
**Age** (years)	53.4±6.9	52.8±7.4	54.3±5.8	0.304
**Gender** (male)	53.0%	55.6%	48.7%	0.504
**Race/Ethnicity**				
African American	66.0%	57.6%	42.4%	**0.015**
Hispanic	29.0%	33.3%	21.6%	0.213
White	28.0%	34.9%	16.2%	0.100
Other	6.0%	6.4%	5.4%	0.603
**Log**_**10**_ **HIV VL (copies/mL)**	1.75±0.33	1.78±0.32	1.70±0.36	0.549
**Undetectable HIV VL (<20 copies/mL)**	78%	69.7%	30.3%	0.207
**Suppressed HIV Viral Load (20–200 copies mL)**	22%	54.5%	45.5%	0.142
**Duration of HIV** (years)	17.2±7.3	18.0±7.2	15.6±7.1	0.150
**CD4 cell count** (cells/μL)	641.7±354.1	633.4±305.6	655.9±428.5	0.780
**Cigarette smoker**	47.0%	36.5%	64.9%	**0.006**
**Alcohol** (drinks/week)	2 (0, 5)	1 (0, 3)	2 (0, 6)	0.284
**BMI** (kg/m^2^)	28.7±5.8	29.0±5.9	28.2±5.6	0.520
**Obese** (BMI>30kg/m^2^)	37.0%	38.1%	35.1%	0.767
**LPS** (EU/mL)	0.59±0.23	0.53±0.24	0.72±0.12	**<0.001**
**I-FABP** (pg/mL)	2515.83± 1072.95	2337.56±1072.95	2866.97±798.06	**0.014**
**sCD14** (pg/mL)	1038.47±424.2	1021.81±402.55	1080.73±480.7	0.552
**sCD27** (pg/mL)	7.95±2.94	7.70±2.52	8.58±3.81	0.291
**sCD163** (pg/mL)	635.71±418.52	613.25±431.73	692.74±385.01	0.393
**hs-CRP** (mg/L)	3.70±3.57	3.58±3.63	3.94±3.48	0.638
**TNF-ɑ** (pg/mL)	8.02 (5.76–11.44)	8.31 (5.78–11.44)	7.48 (3.05–11.47)	0.445
**IL-6** (pg/mL)	0.7 (6.6–50.8)	24.8 (8.5–55.5)	4.9 (3.3–28.3)	0.453

**Bold** indicates statistical significance at P < 0.05.

^a^Data are summarized as mean ± standard deviation (or median [interquartile range]) for continuous variables and No. (%) for categorical outcomes.

^b^Between group comparisons consisted of T-tests (or Wilcoxon Rank-Sum Test) for continuous variables and Chi-square Tests for categorical variables.

### Correlations between biomarkers

As shown in Table 2, blood BE, the major metabolite of cocaine in blood, was directly associated with LPS (*rho* = 0.276, P = 0.028), sCD14 (*rho* = 0.274, P = 0.031), and sCD163 (*rho* = 0.250, P = 0.049). I-FABP only correlated with sCD27 (*r* = 0.221, P = 0.034) and LPS only correlated with sCD163 (*r* = 0.422, P<0.001). Plasma levels of sCD14 correlated with sCD27 (*r* = 0.381, P<0.001) and sCD163 (*r* = 0.240, P = 0.021).

**Table 2:** Correlation matrix^a^.

	**BMI**	**hs-CRP** ^b^	**CD4+**	**I-FABP** ^b^	**LPS** ^b^	**sCD14** ^b^	**sCD27** ^b^	**sCD163** ^b^	**BE** ^c^
**Age**	**-0.231** *	0.117	-0.084	0.093	-0.047	0.010	0.191	-0.139	0.014
**BMI**	-	**0.293** **	**0.234** *	-0.186	-0.132	0.183	-0.010	0.197	0.066
**hs-CRP** ^b^	-	**-**	**0.247** *	-0.066	-0.152	0.061	0.036	-0.057	0.065
**CD4+**	-	-	-	-0.022	0.097	-0.058	-0.181	-0.093	-0.109
**I-FABP** ^b^	-	-	-	-	0.123	-0.088	**0.221** *	0.023	0.133
**LPS** ^b^	-	-	-	-	-	-0.079	0.068	-0.084	**0.276** *
**sCD14** ^b^	-	-	-	-	-	-	**0.381** **	**0.240** *	**0.274** *
**sCD27** ^b^	-	-	-	-	-	-	-	**0.377** **	0.125
**sCD163** ^b^	-	-	-	-	-	-	-	**-**	**0.250** *

**Bold** indicates statistical significance at p < 0.05.

^a^Pearman’s correlation, unless otherwise specified

^b^Log-transformed

^c^Spearman’s correlation

*P < 0.05

**P < 0.01

### Gut permeability and microbial translocation

**Fig 1:**
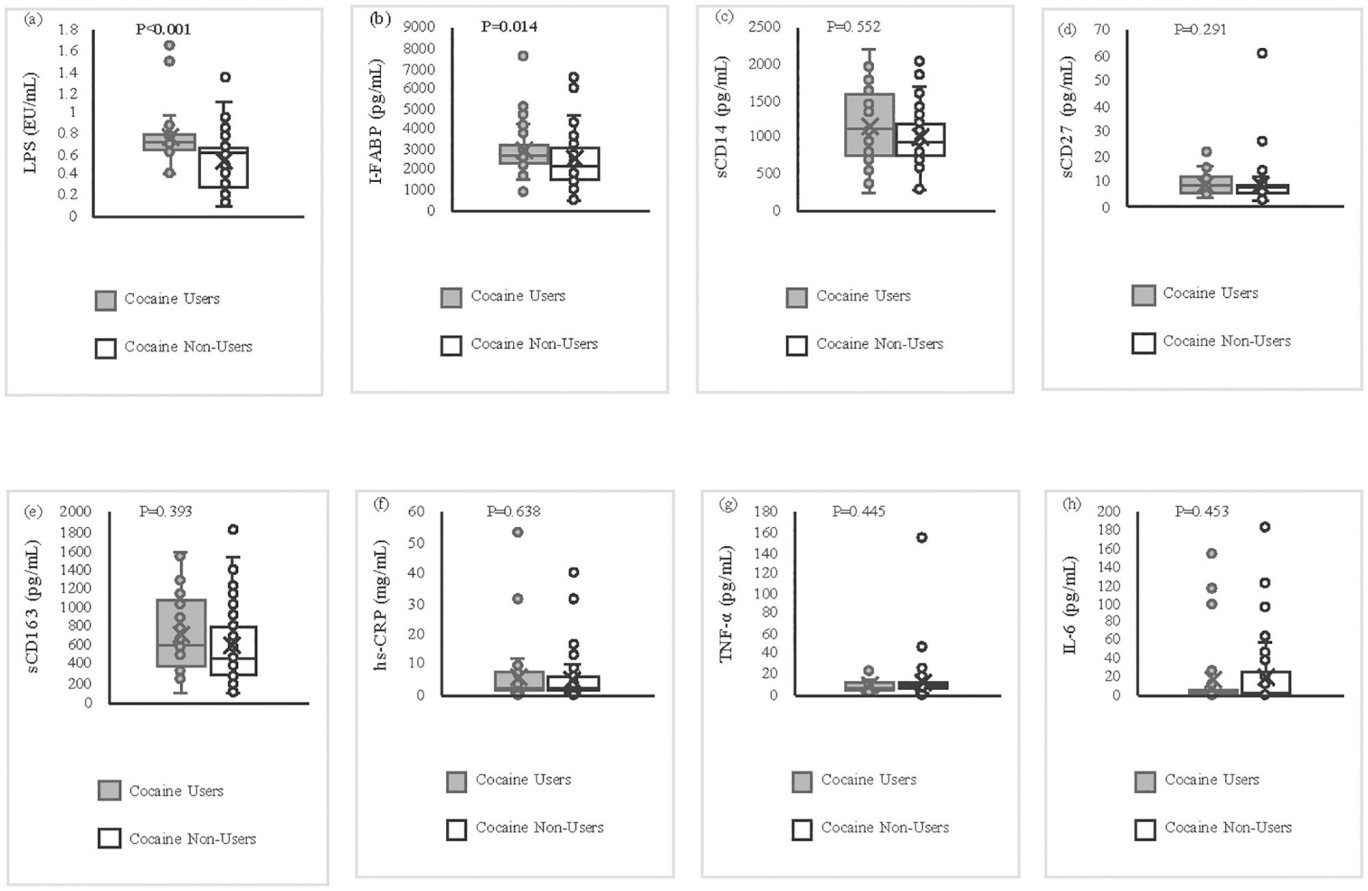
Comparison of gut permeability, microbial translocation, immune activation, and inflammatory markers between cocaine users and non-users. **Bold** indicates statistical significance at P < 0.05. Microbial translocation: a) LPS. Gut permeability: b) I-FABP. Immune activation: c) sCD14, d) sCD27, e) sCD163. Inflammation: f) hs-CRP, g) TNF-α, h) IL-6.

Cocaine users had higher levels of I-FABP (2866.97±798.06 vs. 2337.56±1072.95 pg/mL, P = 0.014) and LPS (0.72±0.12 vs. 0.53±0.24 EU/mL, P<0.001) than cocaine non-users, indicating greater gastrointestinal permeability and microbial translocation among the cocaine users (Fig 1). A series of multiple regression models were carried out to investigate whether cocaine use was associated with I-FABP and LPS levels in plasma, controlling for age, sex, race, CD4 cell count, suppressed HIV viral load (20–200 copies/mL), and cigarette smoking (Table 3). When adjusted for these confounders, cocaine use was significantly associated with both I-FABP (P = 0.033) and LPS levels (<0.001) as shown in Table 3.

**Table 3:** Associations between cocaine use, gut integrity damage, microbial translocation and immune activation.

	I-FABP^a^				LPS^a^				sCD14^a^				sCD27^a^				sCD163^a^			
	B	SD	t	P	B	SD	t	P	B	SD	t	P	B	SD	t	P	B	SD	t	P
Cocaine use	0.24	0.11	2.17	**0.033**	0.39	0.11	3.88	**<0.001**	0.02	0.11	0.19	0.850	0.08	0.09	0.89	0.371	0.44	0.16	2.65	**0.010**
IFABP^b^					0.12	0.11	1.05	0.296	-0.13	0.10	-1.15	0.252	0.16	0.08	1.48	0.142	0.02	0.15	0.24	0.808
LPS^b^									-0.12	0.09	-1.07	0.285	-0.01	0.07	-0.14	0.866	-0.09	0.14	-0.84	0.401

**Bold** indicates statistical significance at P < 0.05.

^a^ Estimates are adjusted for age, sex, race, CD4 cell count, suppressed HIV viral load (20–200 copies/mL), and cigarette smoking.

^b^Log-transformed

Logistic regression analyses indicated that cocaine use was significantly associated with high LPS levels [≥ median 0.63 (IQR:0.48–0.73) EU/mL (P<0.001] after controlling for the same covariate. In addition, cocaine users had 5.15 times higher odds to exhibit high LPS levels than non-users (OR: 5.15 95% CI: 1.89–13.9; P<0.001), as shown in Table 4. No significant association was found between cocaine use and high levels of I-FABP.

**Table 4:** Logistic regression analysis predicting the likelihood of high levels of gut integrity damage and microbial translocation in cocaine users living with HIV.

Variable	Cocaine Users^a^			
	Unadjusted OR (95% CI)	P-value	Adjusted OR^a^ (95% CI)	P-value
**I-FABP (pg/mL)**				
*Above median (≥* 2460.43)	2.69 (0.94–5.11)	0.069	2.20 (0.89–5.45)	0.087
**LPS (EU/mL)**				
*Above median (≥ 0*.*63)*	4.86 (1.95–12.1)	**0.001**	5.15 (1.89–13.9)	**0.001**

**Bold** indicates statistical significance at P < 0.05.

^a^ Reference category non-cocaine users

^b^ Estimates are adjusted for age, sex, race, CD4 cell count, suppressed HIV viral load (20–200 copies/mL), and cigarette smoking.

### Immune activation and inflammation

T-tests showed no differences in markers of immune activation between cocaine users and non-users (Fig 1). Table 3 also shows that there were no significant associations between cocaine use and sCD14 (P = 0.933), sCD27 (P = 0.587) using multiple regression analyses. However, the association between cocaine use and sCD163 was statistically significant at P = 0.010) (Table 3). No significant associations were found between cocaine use and inflammatory markers hs-CRP (P = 0.688), TNF-α (P = 0.433) and IL-6 (P = 0.456) after adjusting for age, sex, race, CD4 cell count, suppressed HIV viral load (20–200 copies/mL) and cigarette smoking. However, LPS levels were significantly associated with hs-CRP (P = 0.040) and TNF-α (P = 0.030) after adjusting for the covariates (S1 Table).

## Discussion

Gastrointestinal complications and persistent immune activation are some of the key pathologies of HIV infection [18, 25]. PLWH are also predisposed to risk factors that may exacerbate these problems, such as substance abuse [28]. In this study, we examined markers of gut permeability, microbial translocation, immune activation and inflammation in the context of cocaine use among PLWH who were on stable ART and virally suppressed. Cocaine users − identified via self-report, urine drug screen, and BE in blood − exhibited greater gut integrity damage and microbial translocation than cocaine non-users, as evidenced by higher levels of I-FABP and LPS, respectively. We also found associations between cocaine use and markers of immune activation, although the results were less consistent. Our data therefore suggest that cocaine use may exacerbate HIV-related microbial translocation and immune activation. These findings emphasize that strategies to prevent, identify, and treat substance use disorders among PLWH are needed and may aid in decreasing the high disease burden in this vulnerable population.

To the best of our knowledge, this is the first study showing direct associations of cocaine use with gut permeability and microbial translocation among PLWH. For example, Volpe et al. [33] did not detect any differences in LPS or sCD14 levels by HIV status or cocaine use, possibly due to a small sample size (N = 32). That said, MASH participants in this study showed similar I-FABP [38–41] and LPS [42–44] levels to the ones reported in other studies conducted in PLWH. Importantly, PLWH display higher concentrations of I-FABP compared to HIV-uninfected peers [38]. Our data show that cocaine users had higher levels of I-FABP and LPS than cocaine non-users. Thus, cocaine use may exacerbate gut permeability and subsequent microbial translocation in PLWH. Interestingly, levels of I-FABP in plasma did not correlate significantly with LPS, sCD14, or sCD163, although others have reported these associations [38]. A novel finding from this study was that cocaine users were 5.15 times more likely to exhibit high microbial translocation levels than non-users, which indicates the damaging effect of cocaine on the GI mucosa.

HIV has been shown to directly affect the enterocytes due to the action of its accessory protein Transactivation of transcription (Tat) that inhibits the uptake of glucose into the enterocyte. In addition, the envelope glycoprotein gp120, promotes calcium accumulation inside the cell affecting the ionic homeostasis [45, 46], and activates the intestinal immune system with the release of pro-inflammatory cytokines [47, 48]. Also, there is a massive loss of CD4+ T cells in the GI lamina propria. This loss also affects the CD4+ T helper 17 cells in the intestine, which are responsible for the intestinal homeostasis, and mucosal regeneration and integrity [2, 3].

Cocaine use has been associated with several gastrointestinal disturbances including diarrhea, nausea, vomiting, and anorexia [33], but mechanisms underlying the impact of cocaine on the gastrointestinal system have not been completely elucidated. While cocaine itself has a relatively short half-life, usually in the range of a few hours, its metabolites remain significantly longer in the circulation [49, 50]. Further, chronic cocaine use extends its half-life, prolonging its elimination from the body [49, 50]. Cocaine may lead to dysbiosis of the gut microbiome and also appears to have the ability to dysregulate the expression of tight junction proteins in the intestinal epithelium, promoting gut permeability of toxic microbial products and subsequent inflammatory response [34, 35]. Thus, the damaging effects of HIV on the enterocytes, the loss of intestinal CD4+ T cells and the likely role of cocaine in the disruption of gut barrier and increased permeability may indicate that cocaine and HIV seem to simultaneously compromise gut homeostasis and further promote microbial translocation. Our data showed that levels of BE in blood correlated with levels of sCD14 and sCD163, suggesting that cocaine metabolites promote immune activation while they remain in blood. However, we did not observe a significant association between cocaine and sCD14 and sCD27 immune activation and inflammatory markers in the regression analyses. It is important to reiterate that all our participants in this study were on stable ART (≥6 months), suppressed viral load (<200 copies/mL), more than half had undetectable HIV viral load (<20 copies/mL), had CD4+ cell count >200 cells/μL, and did not report hazardous drinking (AUDIT score >8). These results indicate that cocaine may directly destabilize gut integrity as evidenced by the association between cocaine use, I-FABP and LPS. In addition, cocaine use was related to immune activation as shown by significant associations with sCD163.

This study had several important strengths, including the inclusion/exclusion criteria, which allowed us to isolate the effect of cocaine use in the context of HIV disease. Indeed, substance use is a risk factor for HIV disease progression [29, 51, 52]. We have previously shown that cocaine use can accelerate HIV disease progression by reducing adherence to HIV treatment, as well as through physiological mechanisms independent of ART adherence [29, 53]. In addition we used urine drug screen and determined cocaine metabolites in blood to strengthen the self-reported data on cocaine use, although even these biomarkers provide a relatively short window of detection [49]. Several limitations of this study should be acknowledged. Due to the eligibility criteria, the results of this study are mainly applicable to PLWH who are virally suppressed and do not exhibit hazardous alcohol consumption patterns. Further, lack of HIV-uninfected participants prevents us from ascertaining how the impact of cocaine differs in PLWH from those uninfected with HIV. To the best of our knowledge, the impact of cocaine on microbial translocation and immune activation has not been examined outside of the context of HIV. Other biomarkers to assess gut permeability including tight junction proteins (occludin, zonulin, etc.), and Regenerating islet-derived protein 3 (REG3α) [8, 54–56] have not been determined in this study. However, LPS employed in this study is a widely used marker of microbial translocation, while I-FABP has been used as a marker of gut permeability in other studies with PLWH [38–41]. Longitudinal studies are needed since the cross-sectional design of this study prevents any determination of a temporal or causative association. Future studies should include participants who use and do not use cocaine, as well as people uninfected with HIV, as this may elucidate the specific changes in gut permeability, microbial translocation, immune activation and inflammation associated with cocaine use in PLWH.

## Conclusions

Cocaine use is considered a relevant factor that accelerates HIV disease progression and promotes mortality among PLWH. The findings of this study suggest that cocaine use is associated with a loss of gut integrity and increased microbial translocation and immune activation in PLWH despite ART use and viral suppression. Thus, cocaine appears to have profound effects on both the gastrointestinal tract and the immune system. Further research with longitudinal design is needed in order to elucidate mechanisms behind the effects of cocaine on gut health and immunity in the context of HIV infection. This study provides important data to aid in the development of effective approaches that target lifestyle modifications and treatment for substance use disorders to decrease the burden of disease among PLWH.

## Supporting information

S1 Table Associations between cocaine use, gut integrity damage, microbial translocation and inflammation. **Bold** indicates statistical significance at P < 0.05. a Estimates are adjusted for age, sex, race, CD4 cell count, suppressed HIV viral load (20–200 copies/mL), and cigarette smoking. bLog-transformed.
(DOCX)

S1 Dataset (XLSX)
