# Supplementary material for: Cocaine use associated gut permeability and microbial translocation in people living with HIV in the Miami Adult Study on HIV (MASH) cohort
Source: PLoS One. 2022 Oct 10;17(10):e0275675. doi: 10.1371/journal.pone.0275675 (PMC9550062; doi:10.1371/journal.pone.0275675)
Supplement: S1 Table — Bold indicates statistical significance at P < 0.05. a Estimates are adjusted for age, sex, race, CD4 cell count, suppressed HIV viral load (20–200 copies/mL), and cigarette smoking. bLog-transformed. (DOCX) [file pone.0275675.s001.docx]

**Hs-CRP^a^ TNF-*α*a IL-6^a^**

|  | B | SD | t | P | B | SD | t | P | B | SD | t | P |
| --- | --- | --- | --- | --- | --- | --- | --- | --- | --- | --- | --- | --- |
| Cocaine use | 0.07 | 0.22 | 0.31 | 0.753 | -0.11 | 0.21 | -0.53 | 0.597 | -0.02 | 0.50 | -0.20 | 0.838 |
| IFABPb | -0.17 | 0.21 | -0.79 | 0.428 | -0.10 | 0.18 | -0.55 | 0.584 | -0.62 | 0.44 | -1.41 | 0.162 |
| LPSb | -0.23 | 0.20 | -2.09 | **0.040** | 0.26 | 0.17 | 2.20 | **0.030** | -0.14 | 0.45 | -1.22 | 0.224 |

**Bold** indicates statistical significance at P < 0.05.

a Estimates are adjusted for age, sex, race, CD4 cell count, suppressed HIV viral load (20-200 copies/mL), and cigarette smoking.

bLog-transformed
